# Experimental Study of Thermally Damaged Concrete under a Hygrothermal Environment by Using a Combined Infrared Thermal Imaging and Ultrasonic Pulse Velocity Method

**DOI:** 10.3390/ma16031040

**Published:** 2023-01-24

**Authors:** Yi Wang, Jiajie Cui, Jun Deng, Hao Zhou

**Affiliations:** 1 National Engineering Research Center of High-Speed Railway Construction Technology, Central South University, Changsha 410075, China; 2 School of Civil Engineering, Central South University, Changsha 410075, China; 3 Logistic Management Office, Guangdong Pharmaceutical University, Guangzhou 510006, China; 4 School of Civil Engineering, Guangzhou University, Guangzhou 510006, China

**Keywords:** thermal damage, concrete, hygrothermal environment, ultrasonic pulse velocity (UPV), infrared thermal imaging (IRT)

## Abstract

This paper proposes a combined inspection method for thermally damaged concrete under a hygrothermal environment. Experiments were conducted to verify the feasibility of the proposed method. Concrete samples with different water–cement ratios (W/C = 0.3, 0.5, 0.7) and moisture contents (dried, 50% saturated, fully saturated) were exposed to elevated temperatures of 200 °C, 400 °C, 600 °C, and 800 °C for 4 h. After cooling to room temperature, infrared thermal imaging (IRT), ultrasonic pulse velocity (UPV) measurements, and mechanical tests were carried out for the damaged concrete samples. The mechanical behavior of thermally damaged concrete with different degrees of water saturation was examined based on mechanical testing. The results show that water can affect the compressive strength and UPV of concrete under certain circumstances, and the residual strength and the heating temperature of the thermally damaged concrete can be evaluated by IRT and UPV measurements. When 50% saturated concrete specimens with a W/C ratio of 0.3, 0.5, and 0.7 are exposed to 200 °C, 12.6%, 27.4%, and 34.6% increases in normalized compressive strength were observed before dropping to approximately 40% at 800 °C. With various moisture contents, the normalized compressive strength variation can be up to 40% at 400 °C in cases with W/C = 0.5 and 0.7. As for UPV, it generally decreases with the increase in moisture content when the peak temperature is 800 °C. On the contrary, whether concrete is saturated or not, there is little difference in temperature change in IRT detection. To obtain a more precise evaluation of concrete structures, IRT can be used to scan a large area to determine the damaged concrete area and areas suspected to be damaged, while UPV could be used to detect concrete members in suspected areas after the completion of IRT scanning.

## 1. Introduction

Fire disasters are one of the most serious problems for reinforced concrete (RC) structures because of the decomposition of cementitious materials and cracking of concrete. When concrete is exposed to elevated temperatures, the mechanical performance of concrete structures can be degraded due to damage [1,2]. To ensure the safety of concrete structures suffering from fire, an evaluation of concrete structures is of great importance, and countermeasures should be taken based on the results of nondestructive detection and evaluation.

For fire-damaged RC structures, there are several conventional detection methods. Before the nondestructive inspection is carried out, spot observation and destructive testing methods are used to detect the fire-damaged RC structures. First, the inspectors enter the spot to observe the environment as well as the damaged RC structures and take some samples from the damaged RC structures. According to the residues left in the environment, the scope of the peak temperatures can be assessed. After that, samples are used for strength testing by the testing organization, and the evaluation of fire-damaged structures is presented [3]. However, due to the complexity of the environment, it is difficult to evaluate the performance of RC structures after fire disasters by spot observation and destructive testing methods, which decreases efficiency and threatens inspectors’ safety. Therefore, to improve inspection efficiency as well as the inspectors’ safety, nondestructive inspection methods are carried out for concrete structure evaluation; yet, some of the nondestructive inspection methods are difficult to use effectively in the fire-damaged area. In a previous study, X-ray scans [4] and piezoelectric ceramics [5,6] were used to determine the internal damage of concrete. However, because of the inconvenience of operation, few inspection results for X-ray scans and piezoelectric ceramics have been presented. In contrast, ultrasonic pulse velocity (UPV), which has the advantage of high accuracy, is becoming one of the most popular nondestructive inspection methods [7,8,9,10,11,12]. Many researchers have studied this method. Yamada et al. [13] studied normal concrete by using UPV before and after fire damage, and the relationship between frequency and concrete strength was analyzed. Lin et al. [14] and Yang et al. [15] further analyzed the relationship between normalized velocity and normalized concrete strength. More importantly, Hou et al. [16] discovered that the relationship between the strength and velocity of ultrasonic waves is clearer than that between the strength and frequency of ultrasonic waves in experiments for thermally damaged reactive powder concrete. After that, the velocity becomes a key factor for UPV analysis. In addition, a range of investigations on fire-damaged new concrete and ordinary concrete have been carried out [17,18,19,20,21,22]. Hager et al. [23] concluded that the UPV of thermally damaged high-performance concrete is higher than that of ordinary concrete, although the trends are similar to those for temperature. In addition, fire-damaged concrete samples with other supplementary materials have been used for UPV inspection, such as crushed rock dust [24], silica fume [25,26,27], solid waste [28], recycled aggregates [29], and thermally damaged self-consolidating concrete samples [30,31]. The results prove that UPV-based inspection can be used to detect fire-damaged concrete and quantitatively evaluate the degree of damage.

However, when the damaged area is quite large, the UPV inspection method has difficulty detecting all the damaged concrete members in a short period of time. Therefore, to improve inspection efficiency, an additional inspection method should be combined with UPV inspection. It is worth mentioning that infrared thermography (IRT) also has great potential and has been widely used to scan concrete damage based on changes in thermal conductivity [32,33]. At present, some investigations with IRT inspection of concrete heat characteristics, such as lightweight concrete [34] and waste glass concrete [35], have been presented. Sun et al. [36] applied IRT to detect the heat performance of 3D-printed concrete. Based on these experimental results, Zhang et al. [37] set up an investigation on IRT detection of fire-damaged concrete up to 900 °C and presented two linear relationships between average temperature change, residual compressive strength, and fire temperatures. In addition, Du et al. [38,39,40] carried out a series of studies with different types of concrete by using IRT, and satisfactory results were obtained. It appears that IRT is effective in evaluating damage caused to building materials [41].

In practice, a variety of testing methods have been chosen to evaluate damaged concrete structures [3,4]. The UPV inspection method has been used frequently to detect damaged concrete, having the advantages of high precision and nondestruction of concrete structures [42]. Nevertheless, the area of a fire disaster is typically vast enough that it is inefficient to only use UPV detection. As a result, adding a supplementary inspection method is of great importance. The IRT inspection method is a good option because it is highly efficient and can be used to scan a large area in a short time. To improve the efficiency and accuracy of the evaluation, IRT inspection can be used for detection first, followed by UPV inspection. On the other hand, the moisture content of concrete is changed frequently under hygrothermal environments. When concrete structures under hygrothermal environments are exposed to fire, the moisture content in concrete will play an important role in the fire damage due to the combined effects of vapor pressure and thermal conductivity difference, and affect the accuracy of damage detection. Therefore, to evaluate the thermally damaged concrete more precisely, moisture content should be a significant factor in this investigation.

From the above discussions, this paper presents a combined inspection method based on UPV and IRT for thermally damaged concrete. To determine the effect of the hygrothermal environment, concrete samples composed of ordinary concrete with varying water-to-cement ratios and moisture contents were used. After experimental procedures of elevated heating, nondestructive inspection, and compression tests, the results of UPV, IRT, and strength evaluation of damaged concrete suffering from elevated temperatures were analyzed and compared with those obtained for undamaged concrete samples. Finally, the limitations of the combined inspection method with IRT and UPV are discussed.

## 2. Experimental Program

**Figure 1:**
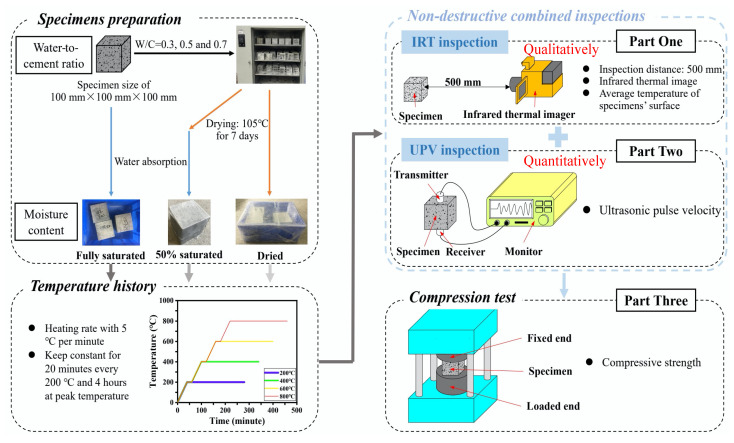
Flowchart for this experimental study.

The overall experimental program is illustrated in Figure 1. Cubic concrete samples with different water-to-cement (W/C) ratios were conditioned with different moisture contents before being exposed to elevated temperatures. Then, both the IRT and UPV methods were implemented to inspect the possible damage within the heated exposed samples. After that, axial compression tests were conducted to evaluate the change in the concrete compressive strength under different hygrothermal and thermal conditions. A detailed experimental program is presented in the subsequent sections.

### 2.1. Specimen Preparation

In this study, a total of one hundred thirty-five cubic concrete specimens were prepared. For comparison, another nine normal concrete specimens, nine mortar, and nine cement paste specimens were cured in the ambient environment. All the specimens have the same dimensions of 100× 100 × 100 mm. When casting the specimens, ordinary Portland cement that was provided by the Guangzhou Shijing Cement Plant (Guangzhou, China) was used. The density of the cement was 3.07 g/cm^3^. The fine and coarse aggregates were composed of siliceous river sand and natural granite crushed stone, respectively. The maximum size and fineness modulus for the fine aggregate were 5 mm and 2.34, respectively. The water absorption and moisture content were 1.10% and 0.01%, respectively. For the coarse aggregate, the maximum size was 20 mm, and the water absorption and moisture content were 0.07% and 0.008%, respectively. The densities of the fine and coarse aggregates were 2.62 g/cm^3^ and 2.66 g/cm^3^, respectively. Specimens with three W/C ratios were prepared. The detailed mixture designs for the concrete, mortar, and cement-paste specimen are summarized in Table 1. The average density of the concrete was 2360 kg/m^3^ [43].

After casting, the specimens were left in the mold for 24 h at ambient temperature. The top of the specimens was covered with a plastic film to reduce the early cracking caused by water vaporization [44]. Once demolded, all of the concrete specimens were cured in a tank with calcium hydroxide solution to obtain full saturation. After 28 days of curing, the samples were divided into three groups based on former experimental investigations [45]. One group was still kept in a tank named fully saturated, while the other two groups of concrete samples were dried for another 7 days at 105 °C. The specimens were weighed to ensure that they were fully dried. After drying, half of the dried samples were sealed to avoid vapor absorption, namely, the dried specimens. The other half of the specimens were immersed in water until the water absorption was 50% of that of fully saturated specimens, namely, 50% saturated in this study [45].

### 2.2. Temperature History

In this study, 5 heating conditions, i.e., 200 °C, 400 °C, 600 °C, and 800 °C, were considered [22]. The dried, 50% saturated, and fully saturated specimens were treated in each heat exposure condition, and three samples were prepared for each case. In addition, specimens treated at 20 °C were employed as a reference test. For the heating treatment, the temperature change was controlled to be 5 °C/min. To avoid explosive concrete spalling while heating, the temperature was kept unchanged for 20 min for every 200 °C step increase in temperature so that a uniform temperature within the specimens could be attained. After reaching the target temperature, the specimens were further conditioned for 4 h at the same temperature [46]. After finishing heating, the concrete samples were removed from the furnace and put in the ambient environment for natural cooling. Then, the cooled specimens were sealed to avoid vapor absorption before nondestructive testing.

### 2.3. Nondestructive Testing

**Figure 2:**
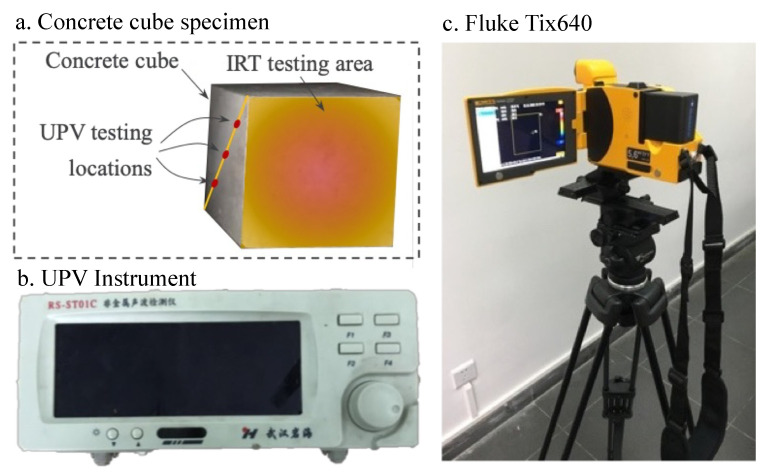
IRT and UPV testing instrument.

As shown in Figure 2, both IRT and UPV were implemented in this study to detect the possible damage caused to the heat-conditioned concrete specimens. The IRT inspection was conducted using a Fluke Tix640, which has a working temperature range between −40 °C and 1200 °C and a thermal sensitivity of 0.03 °C (Figure 2c). In the test, the distance between the specimen and infrared camera was 500 mm, and the sampling frequency was 4 frames per minute. For each specimen, heating and cooling were applied for 2.5 min to take sufficient photos for the subsequent analysis. Once the IRT inspection was completed, the same specimen was tested by UPV at three locations (Figure 2a) with a nonmetallic ultrasonic testing machine obtained from Wuhan Yanhai (Figure 2b). During the UPV test, the receiver and transmitter transducers were attached onto the opposite side of the concrete cube to detect the time and frequency of the ultrasonic waves passing through the concrete cube. Based on these results, the average ultrasonic wave transmitting velocity was calculated and compared to determine the possible damage within the concrete.

### 2.4. Compression Test

To evaluate the mechanical properties of the concrete after heat exposure, an axial compression test was conducted for the concrete specimens. A MATEST 4000 kN testing machine produced by MATEST company (Treviolo, Italy) was employed to complete all the axial compression tests. The compression for the test was controlled at 0.3 MPa per second. After the axial compressive force dropped to 80% of the peak value, the test was terminated.

## 3. Results and Discussions

### 3.1. Mechanical Properties of Concrete under Elevated Temperatures

**Figure 3:**
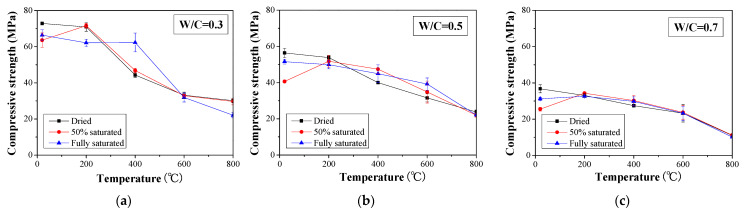
Compressive strength of concrete with different saturation degrees under high temperatures: (**a**) W/C = 0.3, (**b**) W/C = 0.5, and (**c**) W/C = 0.7.

The compressive strength of concrete specimens conditioned in a different environment is presented in Figure 3. As expected, the compressive strength without heating decreases with the W/C ratio when the same moisture content is considered. After heat exposure, a similar trend for the change in compressive strength was found, which is described as follows: when the highest temperature experienced by the concrete specimen is 200 °C, the compressive strength of the dried and fully saturated concrete specimens is slightly decreased compared to the reference specimens. However, an obvious increase in the compressive strength can be observed in the 50% saturated specimens, although its initial strength is lower than that of the other two types of specimens. This trend can even be observed for concrete specimens with W/C ratios of 0.5 and 0.7 at a temperature of 400 °C. Such behavior can be attributed to the vaporization of water in the pores and crystallization in the 50% saturated specimens. Water vaporization, on the one hand, reduces the inner temperature of the concrete so that the chemical bond will not degrade significantly. On the other hand, vaporization and relatively high temperatures can facilitate the chemical reaction of the cement to form more C-S-H products. As such, the overall compressive strength can slightly increase. However, the postcuring effect is less significant in dried specimens conditioned at temperatures higher than 200 °C. For the fully saturated concrete specimens, the vaporized water can apply pressure on the surrounding concrete and thus cause cracking. Therefore, the compressive strength is decreased for both specimens.

**Figure 4:**
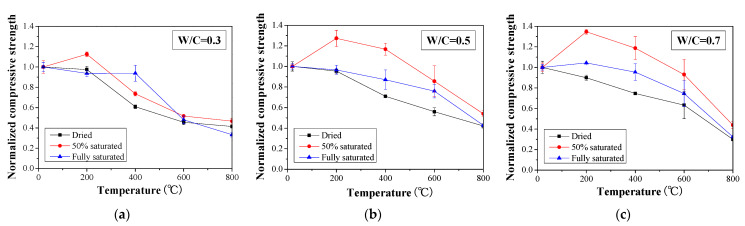
Normalized compressive strength of concrete with different saturation degrees under high temperatures: (**a**) W/C = 0.3, (**b**) W/C = 0.5, and **(c**) W/C = 0.7.

When the temperature is higher than 400 °C, the compressive strength is generally decreased with temperature regardless of the water-to-cement ratio and moisture content (Figure 3). To better illustrate the change in the compressive strength, the compressive strength is normalized with respect to the value obtained for the respective reference specimen for each respective W/C ratio and moisture content (Figure 4). When 50% saturated concrete specimens with a W/C ratio of 0.3, 0.5, and 0.7 were exposed to 200 °C, 12.6%, 27.4%, and 34.6% increases in normalized compressive strength were observed. However, when reaching 800 °C, less than 60% of normalized compressive strength was maintained with respect to each case. The observed drop in compressive strength can be attributed to the occurrence of microcracks and the decomposition of cementitious materials at such high temperatures [47]. In addition, compared to the specimens with a W/C ratio of 0.3, the compressive strength values for specimens with W/C ratios of 0.5 and 0.7 show significant scatter. Nevertheless, the residual compressive strength of all the concrete specimens is approximately 40% of that obtained without heat exposure.

With the above test results, it can be seen that the fire performance of concrete is related to the moisture content within the concrete. As far as the cases investigated in this study, a 50% saturation can benefit the concrete compressive strength when the temperature is below 200 °C. In such a scenario, the postcuring of concrete plays an important role. However, as the heating temperature increases, the concrete compressive strength decreases due to the decomposition of the hydration product [48,49]. There is a large difference for cases with different saturation degrees, especially in cases with W/C = 0.5 and 0.7; the gap reached greater than 40% of the normalized compressive strength at the temperature of 400 °C. Although the strength of fire-damaged concrete under different circumstances could be predicted more precisely with artificial intelligence, such as in a machine learning approach [50,51], large data are necessary to train it, and the results in this study are helpful for the cases under a hygrothermal environment.

### 3.2. Results of Infrared Thermal Imaging

**Figure 5:**
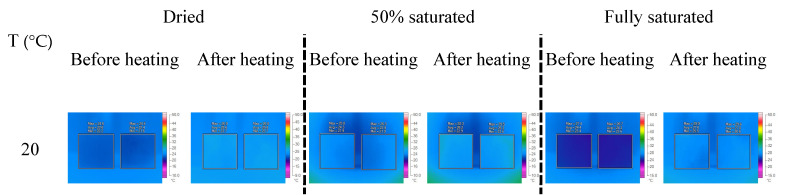

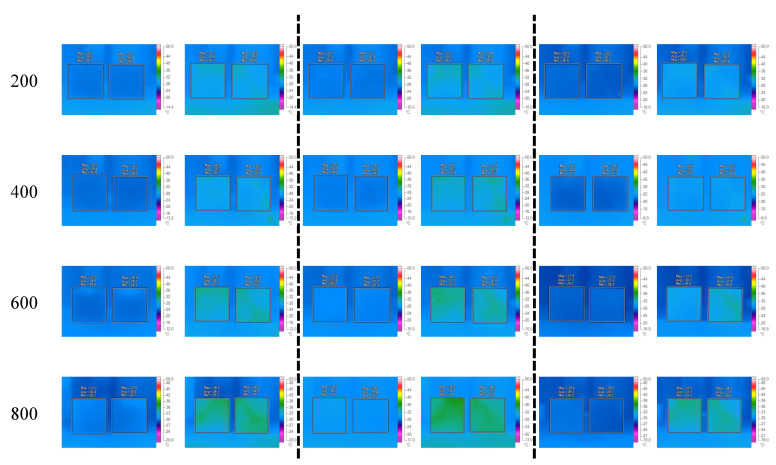
Representative IRT images of the tested concrete specimens with W/C = 0.3.

**Figure A1:**
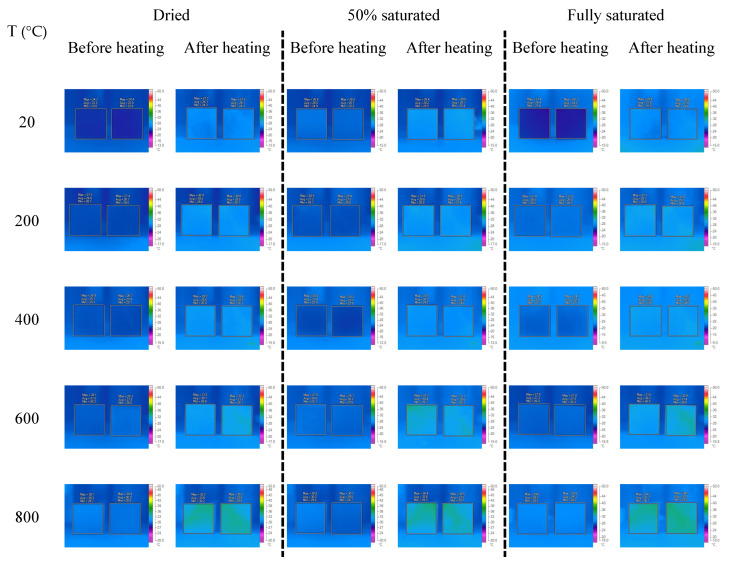
Infrared thermal images of concrete sample, W/C = 0.5.

**Figure A2:**
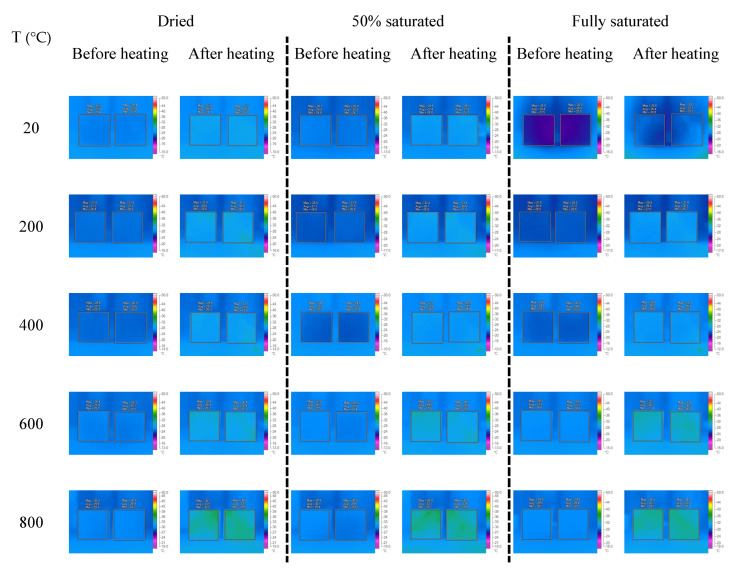
Infrared thermal images of concrete sample, W/C = 0.7.

Representative IRT images before and after heat exposure for concrete specimens with W/C = 0.3 are presented in Figure 5. IRT images for specimens with W/C = 0.5 and 0.7 can be found in the Figure A1 and Figure A2. When the maximum temperature applied is lower than 400 °C, the temperature contour for the dried concrete surface after and before heat exposure is relatively uniform, although the temperature is slightly increased. When the concrete specimens with W/C = 0.3 are heated to 600 °C, a fraction of the concrete surfaces show a significant temperature elevation. As the temperature is further increased to 800 °C, the area with higher temperatures after IRT scanning expands. Especially in the 50% saturated specimens, the temperature contour is more uniform than that in the dried and fully saturated specimens. A similar trend can be found for specimens with W/C = 0.5 (Figure A1). However, for specimens with W/C = 0.7, a significant residual temperature after the IRT test occurs only when the maximum temperature reaches a value of 600 °C (Figure A2).

To quantitatively analyze the thermal damage based on the IRT inspection results, the average temperature change (ΔT) for the exposed concrete surface in the IRT test process was calculated as follows [47]:

(1) $$\Delta T=T_{a}-T_{0}$$

where $T_{a}$ is the average temperature of the concrete surface during the IRT inspection and $T_{0}$ is the initial concrete surface temperature.

**Figure 6:**
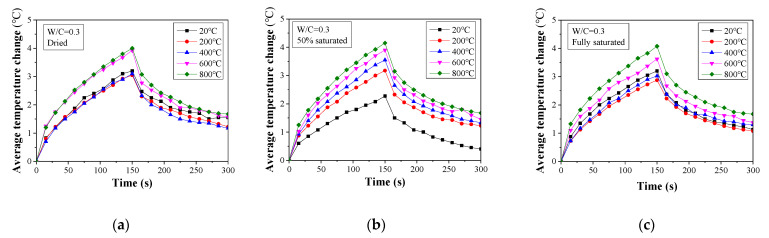
Average temperature change for damaged concrete (W/C = 0.3) determined from thermal image analysis: (**a**) dried, (**b**) 50% saturated, and (**c**) fully saturated.

**Figure 7:**
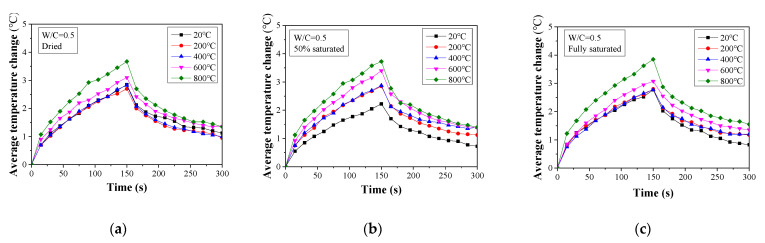
Average temperature change for damaged concrete (W/C = 0.5) determined from thermal image analysis: (**a**) dried, (**b**) 50% saturated, and (**c**) fully saturated.

**Figure 8:**
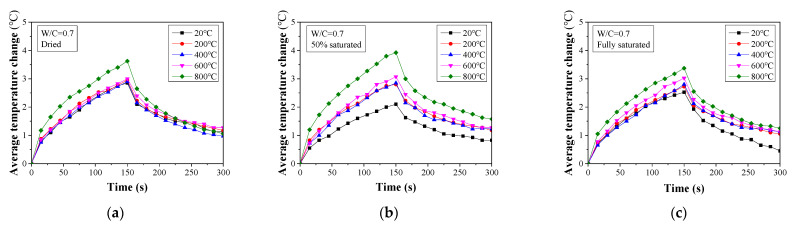
Average temperature change for damaged concrete (W/C = 0.7) determined from thermal image analysis: (**a**) dried, (**b**) 50% saturated, and (**c**) fully saturated.

The temperature change history for all the tested concrete specimens is presented in Figure 6, Figure 7 and Figure 8. When W/C = 0.3, the surface temperature for the dried specimens increases at a temperature over 600 °C. Below this value, the temperature change curves for the specimens are almost identical. A similar trend can also be found in the fully saturated specimens. However, for the 50% saturated specimens, the temperature change history is more distinguishable between specimens with different heat exposures. This observation is consistent with the compressive strength change presented in the previous section. The decomposition of the cement hydration product in the dried specimens exposed to temperatures higher than 400 °C and the microcracking caused by the vapor pressure reduces the heat diffusion [48,49]. Consequently, the concrete surface temperature increases significantly. 

**Figure 9:**
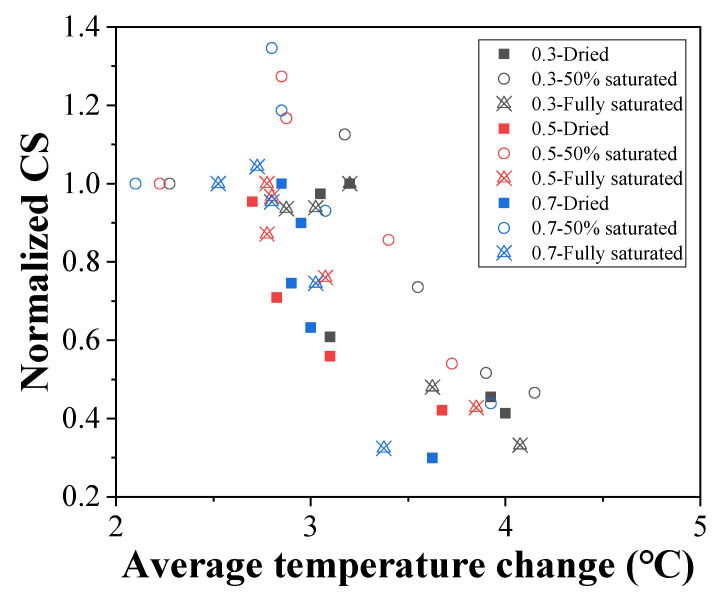
Relationship between the normalized compressive strength and average temperature change in all cases.

Combined with the aforementioned results of the IRT inspection and strength testing, the correlation of average temperature change and strength for thermally damaged concrete is presented in Figure 9. In the cases of dried and fully saturated concrete, there was the least average temperature variation when the residual compressive strength of thermally damaged concrete was over 60% of the original one. Afterwards, a drastic drop occurred since concrete residual strength became less than 60%. Compared with the dried and fully saturated cases, when concrete was in half saturation, the normalized compressive strength was slightly higher, which can agree with the mechanical performance. Therefore, it can be concluded that IRT inspection can be used to qualitatively determine the damage caused to concrete subjected to elevated temperatures.

### 3.3. Results of UPV Testing

**Figure 10:**
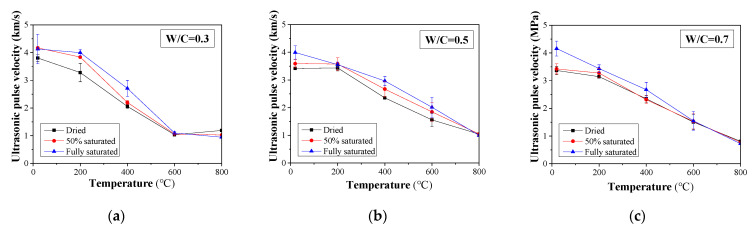
UPV change for damaged concrete after heat treatment with different saturation degrees: (**a**) W/C = 0.3, (**b**) W/C = 0.5, and (**c**) W/C = 0.7.

**Figure 11:**
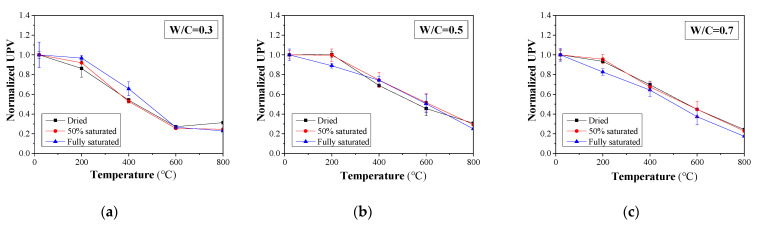
Normalized ultrasonic pulse velocity for concrete with different moisture contents: (**a**) W/C = 0.3, (**b**) W/C = 0.5, and (**c**) W/C = 0.7.

**Figure A3:**
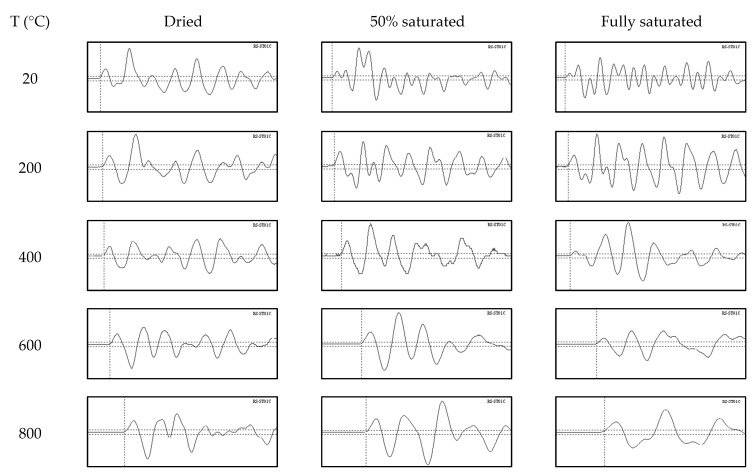
Ultrasonic waves for concrete in different cases, W/C = 0.3.

**Figure A4:**
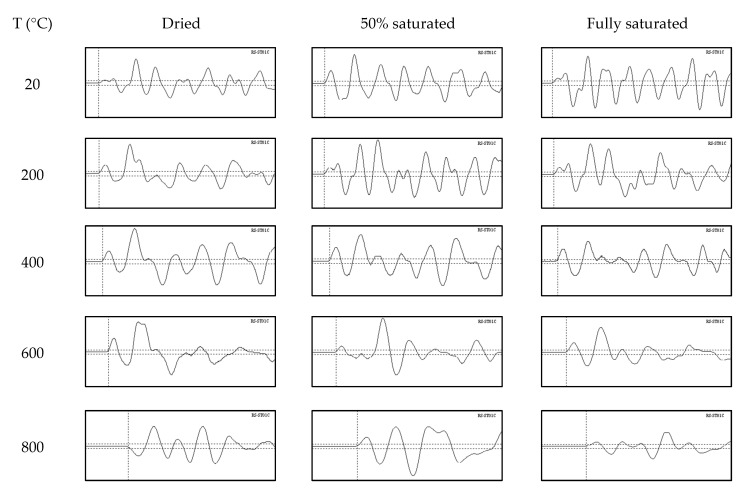
Ultrasonic waves for concrete in different cases, W/C = 0.5.

**Figure A5:**
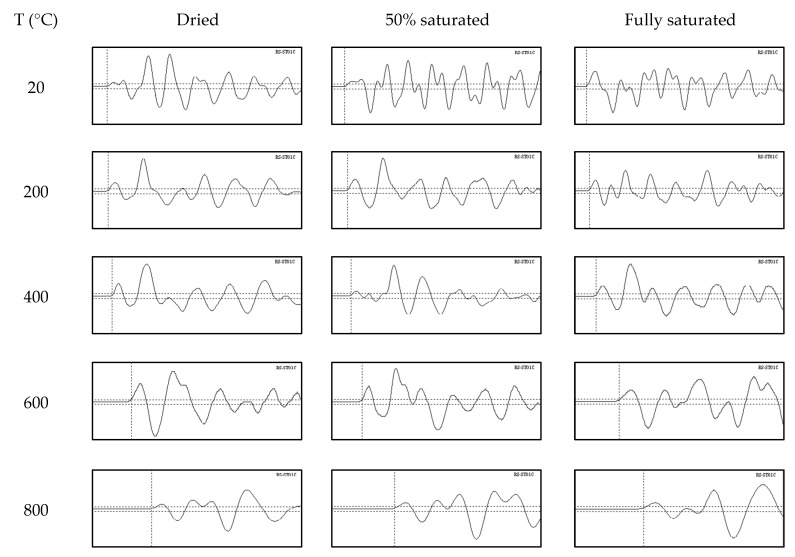
Ultrasonic waves for concrete in different cases, W/C = 0.7.

When concrete structures suffer fire damage, microcracks are formed at the bonding interface between the aggregate and mortar as well as the concrete surface due to thermal expansion and the decomposition of cementitious materials. As such, the velocity of the ultrasonic pulse is reduced [48]. The UPV and normalized UPV of concrete samples with different W/C ratios exposed to different temperatures are presented in Figure 10 and Figure 11, respectively. Ultrasonic waves for concrete in different cases can be found in the Figure A3, Figure A4 and Figure A5. Generally, UPV is increased with the moisture content. This might be attributed to the fact that UPV in water is greater than that in air. In addition, the UPV refraction coefficient between water and concrete is greater than that between air and concrete. As such, when a high level of moisture is presented, the resultant UPV in the multiphase body of concrete can be achieved. Moreover, it can be observed that when W/C = 0.3, the UPV of concrete samples with different moisture contents shows a slight reduction for a temperature below 200 °C. Such a trend can also be observed in dried and 50% saturated concrete specimens with W/C = 0.5 and 0.7. For the fully saturated specimens, a significant reduction in UPV is recorded. Such behavior can be explained as follows: when W/C = 0.3, the fully saturated specimens will experience a postcuring, and that part of the moisture within the concrete will turn into hydration products; thus, the concrete microstructure will become denser. Although part of the existing hydration product may decompose, the resultant effect is that the elasticity of concrete will not change significantly, and thus, the UPV will change. However, in the scenario that the W/C is greater than 0.3 and the specimens are fully saturated, the excessive water within the concrete will turn into vapor and leave voids in the concrete. As such, the transmission of the ultrasonic pulse slows down.

**Figure 12:**
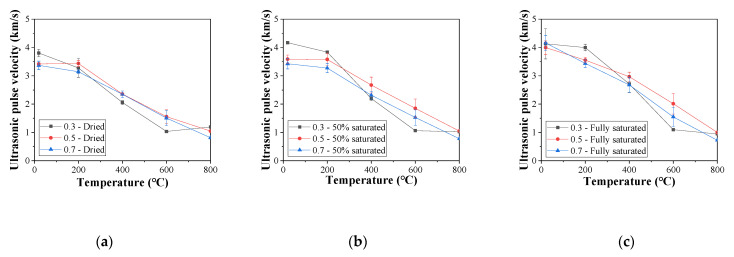
Ultrasonic pulse velocity for concrete with different moisture contents: (**a**) dried, (**b**) 50% saturated, and (**c**) fully saturated.

To further investigate the influence of the W/C ratio on the UPV in concrete subject to elevated temperatures, the UPVs of specimens with all the saturation conditions are summarized in Figure 12. At ambient temperature, UPV is decreased as the W/C ratio is increased for the dried and 50% saturated concrete specimens (Figure 12a,b). However, the W/C ratio is observed to have little influence on the UPV if the specimen is fully saturated. This difference in UPV can be attributed to the microstructure difference for concrete with varying W/C ratios. When the samples are not fully saturated, a lower W/C ratio usually leads to a denser microstructure, which can lead to a higher UPV. For fully saturated specimens, the voids are filled with water, and the influence of the voids becomes less significant.

When the concrete specimens are exposed to temperatures above 200 °C, the concrete specimens with W/C = 0.3 show an almost linear decrease in UPV, and the decreasing rate is much greater than that of the other two types of concrete. This can be attributed to the fact that denser concrete is prone to cracking due to high internal pressure. Therefore, more microcracks within concrete will occur under elevated temperatures, and the UPV will decrease faster. In addition, the reduction rate for the UPV in concrete specimens with W/C = 0.3 slows down when the exposed temperature reaches 600 °C, indicating that the microcracks within the concrete become stable. Such a change in the UPV reduction rate is absent in the other two types of concrete specimens. Moreover, for the dried specimens, the difference in the UPV of concrete with different W/C ratios is much less significant than that for the saturated specimens.

With the above discussion, it can be briefly concluded that the UPV of concrete drops as temperature increases, regardless of the W/C ratio and moisture content. As UPV mainly reflects the elastic property of the material that is exploited to transmit the ultrasonic pulse, a decrease in the UPV indicates a degradation of the modulus of the concrete suffering from exposure to high temperatures caused by fire. It should be noted that although the concrete modulus deteriorates when exposed to elevated temperatures, the concrete strength can increase in certain cases, as discussed in the previous section. This is justified by the fact that the concrete modulus is related to the microcracks formed within the concrete, while the concrete strength is more related to the macrocracks. A decrease in the concrete modulus does not necessarily entail a drop in the concrete strength.

**Figure 13:**
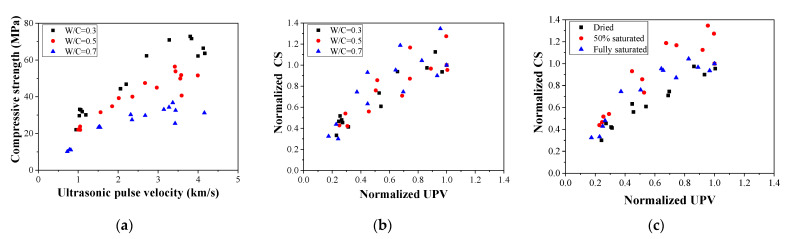
Relationship between compressive strength and ultrasonic pulse velocity: (**a**) average value, (**b**) normalized value–W/C, and (**c**) normalized value–saturation degree.

**Figure 14:**
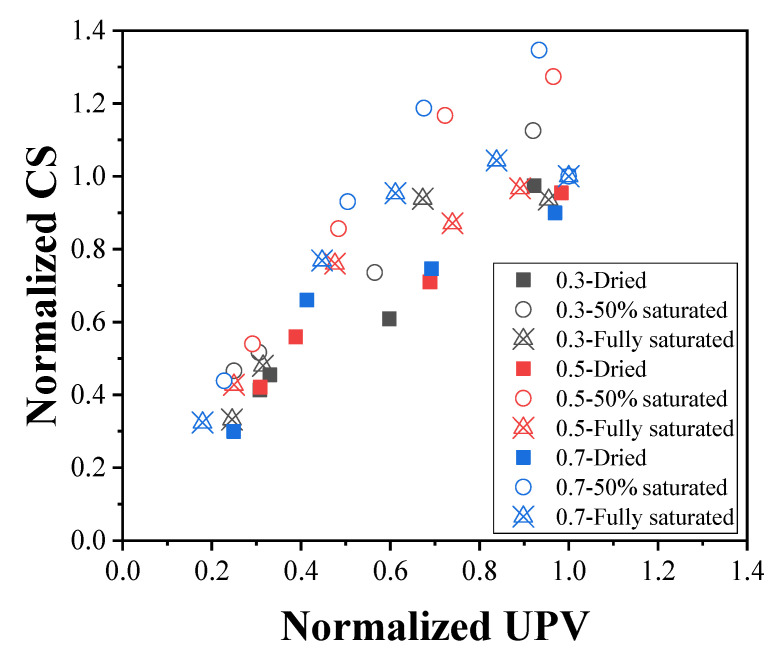
Relationship between the normalized compressive strength and average temperature change in all cases.

According to the UPV and compressive strength results, the relationship between the strength and UPV can be discussed, which is illustrated in Figure 13 and Figure 14, respectively. When the moisture content and W/C ratio factors are separated (see Figure 13), it is observed that the performance of the concrete can hardly be quantitatively determined without normalization. As shown in Figure 13a, when the UPV is over 2 km/s, the compressive strength values are scattered, which makes it difficult to analyze the data accurately. Therefore, the UPV and compressive strength data shown in Figure 13a were normalized based on prior research [15]; in this way, all the points for various cases can be gathered in a certain area (see Figure 13b). On the other hand, 50% saturated concrete shows higher performance than dried and fully saturated concrete in terms of normalized UPV and compressive strength (see Figure 13c). When the moisture content and W/C ratio factors are mixed (see Figure 14), it is observed that the performance of the concrete can be quantitatively determined from the UPV. Consequently, UPV can be used to evaluate concrete strength after fire damage.

### 3.4. Discussion for a Combined Ultrasonic Pulse Velocity and Infrared Thermal Image Detection Method

According to the results of IRT–normalized compressive strength (see Figure 9), when the average temperature variation was around 3 °C, the normalized compressive strength ranged from 0.6 to 1.4, which had an unpredictable influence on the structural safety. To ensure the accuracy of structural safety via nondestructive inspection, a quantitative detection method should be added to support IRT. In 1966, Whitehurst [52] set up criteria for the evaluation of concrete quality by UPV, which are presented in Table 2. Combined with the UPV detection results obtained from this experiment, when concrete is exposed to 200 °C, its quality is affected by the moisture content. However, according to the experimental results, all the concrete specimens become poor under a peak temperature of over 400 °C. The concrete quality can be divided into three different parts based on the UPV, with borderlines at 3.0 km/s and 3.5 km/s, respectively.

However, when the UPV ranges between 3.0 km/s and 3.5 km/s, the concrete quality is doubtful and becomes difficult to evaluate. Consequently, it is of great importance to evaluate the normalized UPV when concrete quality is doubtful. The normalized UPV was calculated using Equation (2) [23]:

(2) $$\omega_{C}=\frac{v_{D}}{v_{C}}$$

where $\omega_{C}$, $v_{D}$, and $v_{C}$ represent the normalized UPV, damaged normal concrete UPV, and undamaged normal concrete UPV, respectively. Regarding $v_{C}$, Lin et al. [12] proposed two equations, Equations (3) and (4), for calculating the concrete UPV based on the volume change in mixture proportions:

(3) 1vC=VpastevP+(1−Vpaste)×SAvFA+(1−Vpaste)×(1−SA)vCA

(4) 1vC=Vpaste+(1−Vpaste)×SAvM+(1−Vpaste)×(1−SA)vCA

where $v_{P}$, $v_{FA}$, and $v_{CA}$ represent the UPVs of cement paste, sand, and stones, respectively; $V_{paste}$ and SA represent the volume of cement paste and the sand–aggregate ratio, respectively, which are based on the mixture proportions (see Equations (5)–(6)) [44].

(5) $$\frac{m_{CE}}{\rho_{CE}}+\frac{m_{FA}}{\rho_{FA}}+\frac{m_{CA}}{\rho_{CA}}+\frac{m_{W}}{\rho_{W}}=1$$

(6) SA=mFAmFA+mCA

where $m_{CE}$, $m_{FA}$, $m_{CA}$, and $m_{W}$ represent the mass of cement, sand, stones, and water, respectively; $\rho_{CE}$, $\rho_{FA}$, $\rho_{CA}$ and $\rho_{W}$, and represent the density of cement, sand, stones, and water, respectively. However, another equation can be used for calculating the mixture proportions, which is shown in Equations (7)–(8) [44].

(7) $$w_{C}=w_{W}+w_{CE}+w_{FA}+w_{CA}$$

(8) SA=wFAwFA+wCA

where $w_{C}$ is the normal concrete weight in the ambient environment per cubic meter and $w_{W}$, $w_{CE}$, $w_{FA}$, and $w_{CA}$ represent the weights of water, cement, sand, and stones per cubic meter, respectively. In engineering practice, concrete mixture proportions are usually presented in mass per unit volume for each component material (see Equations (7)–(8)) rather than by the volumes of composite materials. Therefore, for convenience of application and to increase accuracy, the density of each composite material was considered. Equations (3)–(4) can be transformed into Equations (9)–(10).

(9) wCvC=wW+wCEvP+(wC−wW−wCE)×SAvFA+(wC−wW−wCE)×(1−SA)vCA

(10) wCvC=wW+wCE+(wC−wW−wCE)×SAvM+(wC−wW−wCE)×(1−SA)vCA

**Figure 15:**
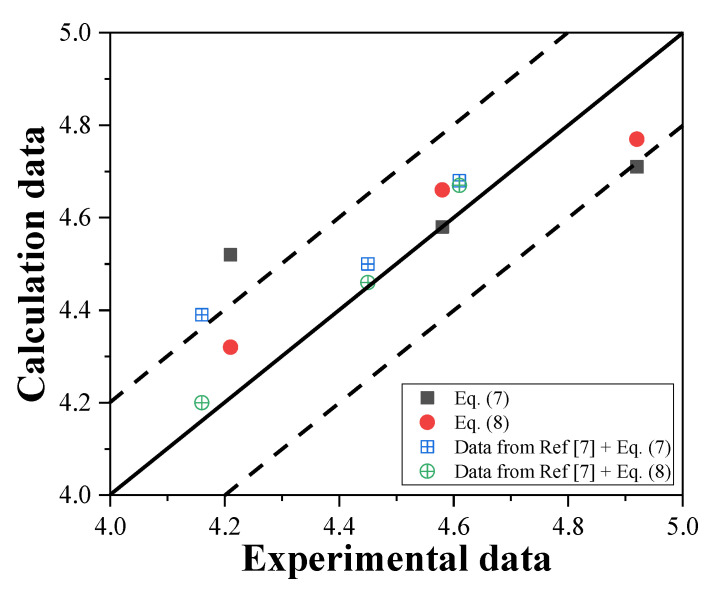
Comparison of the calculation and experimental data.

The results obtained from the application of these equations are presented in Figure 15. It is observed that the calculation data are mostly similar to the experimental data. This result indicates that both Equations (9) and (10) can be used to calculate the UPV under different mixture proportions. However, the UPV of stones in the experiment is smaller than that of sand because of the irregular shape of the stones. Therefore, further discussion on UPV inspection should be considered.

Based on the results shown in Figure 14, when the normalized UPV value is less than 0.8, the normalized compressive strength is usually less than 1.0. Therefore, it can be said that when the normalized UPV value is less than 0.8, the quality of the concrete can be considered to be poor. In contrast, when the normalized UPV value is more than 0.8, the quality of the concrete can be considered to be good. Thus, when concrete structures are exposed to fire, IRT can be used to scan the area to determine the damaged and doubtful areas. After completing the scanning, UPV can be used to detect concrete members in the doubtful area, which can be used to evaluate the concrete structures more precisely. From the combined inspection method with UPV and IRT, the distraction effect due to moisture content could be largely reduced.

## 4. Conclusions

This paper proposes an effective combined inspection method for the evaluation of fire-damaged concrete based on UPV and IRT techniques. Experiments were carried out to study thermally damaged concrete in a hygrothermal environment and to verify the effectiveness of the proposed nondestructive detection method. From the results and discussions, the following conclusions are reached:

a. Before the temperature reaches 400 °C, the moisture inside concrete can absorb a certain quantity of heat during heating, which can reduce the change in the average surface temperature determined by IRT. For an elevated temperature of over 600 °C, chemical decomposition becomes the key factor affecting the average temperature change determined from IRT inspection. The UPV is found to generally increase with moisture content for exposure to temperatures above 600 °C but decreases with exposure to temperatures below 600 °C. In contrast, at 800 °C, the UPVs for the fully saturated specimens are lower than those obtained for the dried and 50% saturated specimens.
b. Suffering from thermal damage, the compressive strength of concrete is gradually decreased with elevated temperatures but not in a monotonic manner. Instead, for fully saturated concrete, the compressive strength remains almost unchanged until 400 °C. In particular, at 200 °C, the concrete strength in 50% saturated cases under W/C ratios of 0.3, 0.5, and 0.7 is 12.6%, 27.4%, and 34.6% higher than that of the control group due to the postcuring effect, which verifies the results obtained from characterization by IRT and UPV.
c. IRT can be used to distinguish damaged concrete areas and suspected areas, while UPV can be further used to evaluate the performance of concrete in suspected areas, which can lead to more efficient inspection of fire-damaged concrete. However, the quantitative relationship between UPV, IRT, and compressive strength still requires further investigation.

## Figures and Tables

**Table 1:** Mixture designs for the concrete, mortar, and cement paste.

	W/C	Water (kg/m^3^)	Cement (kg/m^3^)	River Sand (kg/m^3^)	Crushed Stones (kg/m^3^)	S/A(%)
Concrete	0.3	205	683	589	883	40
	0.5	205	410	611	1134	35
	0.7	205	293	745	1117	40
Mortar	0.3	205	683	589	-	-
	0.5	205	410	611	-	-
	0.7	205	293	745	-	-
Cement paste	0.3	205	683	-	-	-
	0.5	205	410	-	-	-
	0.7	205	293	-	-	-

**Table 2:** Classification of concrete quality according to ultrasonic pulse velocity [49].

UPV (km/s)	Concrete Quality
>4.5	Very good
3.5—4.5	Good
3.0—3.5	Doubtful
2.0—3.0	Poor
<2.0	Very poor

## Data Availability

Data will be made available on request.
